# Urinary strong ion difference and acute kidney injury: an early marker of renal dysfunction?

**DOI:** 10.1186/cc14441

**Published:** 2015-03-16

**Authors:** P Balsorano, A De Gaudio, Stefano Romagnoli, Ipsita Krishnan

**Affiliations:** 1 AOUC Careggi, Florence, Italy; 2 Rhode Island Hospital, Providence, RI, USA

## Introduction

Kidneys play a crucial role in the regulation of electrolytes and acid-base homeostasis. Impaired renal function is associated with greater urinary strong ion difference (SIDu) in patients with metabolic acidosis [1]. In critically ill patients, several factors, such as infused fluids and acid endogenous production, would lead to changes in plasma SID and acid-base homeostasis without renal regulation of urinary electrolytes and SIDu [2]. Hence, AKI can be highlighted as an inability to address acid-base metabolic disturbances, which may be detected before major increases in creatinine or decreases in urine output. We evaluated the effects of renal function on urinary strong ion excretion using the Stewart approach to acid-base in critically ill patients with AKI.

## Methods

A retrospective study was conducted. Patients with a diagnosis of AKI according to KDIGO creatinine criteria and available urinary chemistry at one point during their ICU stay were evaluated. Day 0 was defined as the day when SIDu was calculated from urinary spot analysis (SIDu = Na+U + K+U - Cl-U). Patients were followed and staged for AKI in the next 3 days. AKI reversibility was defined according to the lack of criteria for AKI.

## Results

In total, 143 critically ill patients with a diagnosis of AKI were included. SIDu at day 0 did not differ between different AKI stages at day 0. SIDu at day 0 was statistically different between different AKI stages at days 1, 2, 3 (Table 1). SIDu at day 0 was statistically different between reversible and not reversible AKI at days 1, 2, 3 (Table 2). A conventional receiver-operating curve was generated to assess the accuracy of SIDu to predict AKI reversibility at day 1. AUC for SIDu was 0.82 (*P *< 0.0001; 95% CI: 0.75 to 0.88).

**Table 1:** SIDu (mEq/l) between different AKI stages at days 1, 2, 3 post admission.

AKI stage					
	**3**	**2**	**1**	**0**	***P *value**

Day 1	48.1 (21)	46 (22)	37.9 (20)	17.3 (22)	<0.001
Day 2	40.2 (23)	45.9 (20)	45 (23)	29 (22)	0.004
Day 3	40.3 (26)`	47.2 (18)	53.2 (23)	31 (23)	.006

**Table 2:** SIDu (mEq/l) between reversible versus not reversible AKI at day 1, 2, 3.

	Reversible	Not reversible	*P *value
Day1	16.8 (23)	43.9 (21)	0.0001
Day2	28.5 (24)	45.3 (22)	0.0001
Day3	30 (24)	47.3 (21)	0.0001

## Conclusion

SIDu identified patients with reversible AKI with good accuracy. SIDu can be a promising, simple and cost-effective tool in AKI patient evaluation. Further research is needed to assess SIDu capability to early detect patients with renal dysfunction before increases in creatinine or decreases in urine output.

**Figure 1:**
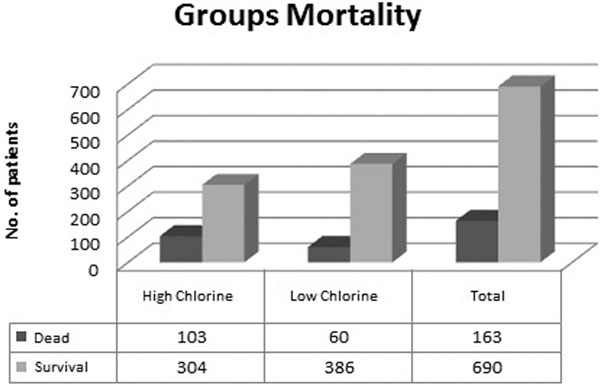
**Group mortality, high and low chlorine**.
